# Developing Metacognition of 5- to 6-Year-Old Children: Evaluating the Effect of a Circling Curriculum Based on Anji Play

**DOI:** 10.3390/ijerph191811803

**Published:** 2022-09-19

**Authors:** Chen Chen, Jianfen Wu, Yunpeng Wu, Xiaoyun Shangguan, Hui Li

**Affiliations:** 1 Jing Hengyi School of Education, Hangzhou Normal University, Hangzhou 311121, China; 2 School of Teacher Education, Dezhou University, Dezhou 253023, China; 3 Shanghai Institute of Early Childhood Education, Shanghai Normal University, Shanghai 200234, China; 4 School of Education, Faculty of Arts, Macquarie University, Sydney, NSW 2109, Australia

**Keywords:** metacognitive training, Anji Play, young children, quasi-experiment, kindergarteners

## Abstract

Metacognition plays an important role in young children’s learning and daily life activities. Based on Anji Play, we designed a metacognition enhancement program named Circling Curriculum for Metacognition Training (CCMT). With a quasi-experimental design, we examined the effects of the CCMT program on the metacognition of 5–6 year old Chinese children. Two classes of 5–6 year old children were randomly assigned into an experimental group (n = 25, 10 girls, mean age = 65.92 months, SD = 3.58) and a control group (n = 22, 10 girls, mean age = 66.77, SD = 3.87). The experimental group received the three-month CCMT, while the control group received routine teaching activities without imposing any interventions. All children took the metacognition test before and after the intervention. Results indicated that (1) there was no significant difference between the experimental group and the control group in all dimensions of metacognitive ability in the pre-test; (2) the experimental group exhibited better metacognitive ability than the control group in most dimensions of metacognitive ability in the post-test; and (3) the gain scores in the metacognitive ability of experimental group were significantly higher than those of the control group. The results are very encouraging and suggest that CCMT can foster the development of the metacognitive ability of young children.

## 1. Introduction

Metacognition is a conscious reflection on one’s thought processes, in which one’s cognitive activity is the object of awareness [1]. Educational psychologists have long promoted the importance of metacognition in regulating and supporting student learning [2]. Anji Play is the abbreviation of the kindergarten game education model that originated in Anji, a county in Zhejiang Province of China, and expanded to most parts of China. It has become an international model of early education, which has been introduced to many countries in Europe, America, and Africa. Anji Play emphasizes the independent play of young children, advocates generating teaching from play, and maximizes children’s learning and development through observation, sharing, and interpretation. It lists “reflection” as one of the five keywords in its play curriculum [3]. Meanwhile, Head Start, the major early childhood development program in the United States, has repeatedly included reflection as the keywords in revising early childhood learning and development standards [4]. The Sutton Trust-EEF Teaching and Learning Toolkit is a teaching and research project initiated by the British government, which is committed to integrating teaching methods with high effect size proved by rigorous experimental data into “The Teaching and Learning Toolkit” to provide accessible evidence-based information for policy-makers and practitioners to inform educational decision-making [5]. Metacognition instruction is considered the best performing strand of the Toolkit. Prior empirical research has shown that children who were effectively taught metacognitive skills tend to make better progress than children who were not taught such skills [6].

Prior studies indicate that metacognition accounts for roughly 17% of a child’s academic achievement, while intelligence accounts for approximately 10%. [7] Teaching and learning metacognitive skills and knowledge can add value to the whole curriculum. Regardless of the course in which metacognitive skills are taught, the students’ performance will improve [8]. After two months of metacognitive intervention, children in grade three improved their metacognitive ability [9]. Meta-analysis of twenty empirical studies suggested that metacognitive instruction is beneficial for children in all grade levels [10]. Metacognition can be successfully taught in different education stages ranging from primary school to university, and it is critical for improving thinking skills [11]. Thus, it is necessary to design a metacognition enhancement program in China and examine its effectiveness to improving the metacognition of young children.

### 1.1. The Structure and Measurements of Metacognition

Metacognition includes two main subcomponents generally referred to as the knowledge of cognition and regulation of cognition [12]. The knowledge of cognition includes knowledge of persons, knowledge of tasks, and knowledge of strategies [13]. (1) The knowledge of persons refers to children’s knowledge of themselves or others as cognitive subjects. (2) The knowledge of tasks includes knowledge of the purpose and the characteristics of the task. (3) The knowledge of strategies refers to knowing which strategy to use in a task and interpreting the strategy (i.e., knowing when to use the strategy, why they are using the strategy, and how to use the strategy).

The regulation of cognition include on-line and off-line components [14]. First, the On-line component includes planning, monitoring, debugging, and strategy selection, and this study focused on the first three. (1) Planning includes choosing the right strategy, predicting before operating, and how to allocate time and attention [15]. (2) Monitoring is the periodic checking of the state of oneself in one’s learning process [12]. (3) Debugging is detecting one’s own mistakes and making timely corrections [16]. Second, the off-line component includes prediction and evaluation: Prediction is making predictions about whether one can complete a task [17] and evaluation is the process by which children use certain criteria to make judgments about their own and others’ behavior and evaluate their own processes and effects [11].

With regard to the assessment of metacognitive abilities, within much of the metacognition literature, there has been an emphasis on using self-reporting methods or rating scales to understand the individuals’ metacognitive processes [18]. The Junior Metacognitive Awareness Inventory is divided into two versions according to age: lower grade and higher grade. The metacognition of children in grades 3–9 is assessed in the form of self-reporting [19]. AA questionnaire was used to assess the general metacognitive and domain-specific metacognitive strategies (i.e., mathematical strategies) by presenting students with a range of strategies and asking them to indicate if and how often they used them by using a 5-point Likert scale ranging from “never” to “always” [20]. However, relying on verbal self-reporting can easily lead to underestimating the metacognitive abilities of young children due to immature verbal development. In previous studies exploring metacognitive processes in young children, there was clear evidence indicating that they were capable of finishing tasks and adapting their behaviors effectively but unable to report verbally on what they had done [21].

Observing the children’s behavior and developing coding frameworks are key methods for assessing the metacognitive abilities of young children. For example, the Strategic Behavior Observation Scale (SBOS) [22], Children’s Independent Learning Development (CHILD 3–5) instrument, and the Cambridgeshire Independent Learning (C Ind. Le) coding framework [7] are widely used. However, the applicability of these assessments has not been tested in China. The Metacognitive Monitoring Task developed by Chinese researchers has been applied in kindergartens in China [23]. Teachers evaluate the regulation of the children cognition level by observing their behavior in a puzzle game. The Metacognitive Knowledge Interview Questionnaire uses teacher evaluation to evaluate the children’s knowledge of cognition level, and its applicability to children aged 5–6 in Chinese public schools has also been supported [24].

### 1.2. Metacognition Interventions in Early Childhood

There is still some controversy over the timing of the development of metacognition. Some researchers consider it as a late-developing capability. A review reported that the picture emerging from much of the literature remained that metacognitive skills emerged at the age of 8–10 years [7].

However, metacognitive behaviors have emerged since early childhood. Children aged three to five exhibited verbal and nonverbal metacognitive behaviors during problem-solving through video-recorded observation including the expression of cognitive knowledge, cognitive regulation, and the regulation of emotional and affective states. This suggests that, in some specific situations, young children can use metacognitive skills to regulate their cognition and behavior based on prior knowledge and experience [25]. Children aged 2 to 2.5 years have certain initial metacognitive forms since they can evaluate what they know and do not know (metacognitive monitoring). Therefore, they start using specific words to describe their states of mind such as “to know”, “to think”, “I know”, and “I do not know” [26]. Children are able to accurately monitor their performance and discriminate their certainty—uncertainty judgment in the age range of 5.5–7.5 [27]. The preschool age is the start time to develop metacognitive structures including the knowledge of cognition and processes [28].

Extensive evidence suggests that educational interventions can improve metacognition [29]. First, a short-term metacognitive intervention (including creating situations, assigning tasks, and asking questions) with grade 3 children indicated that children who received the metacognitive intervention performed better than children in the control group [9]. Second, offering children a proper task and the opportunity to work with others on a problem could help with the children’s development of metacognition, and prompt them to self-reflect, construct new effective strategies, and discard ineffective ones [30]. Finally, the experimental group with third-grade children who received metacognitive training scored significantly higher on the knowledge of cognition and metacognitive skills on the post-test [31]. Research indicated that well-designed metacognitive instruction positively impacts metacognitive behavior and domain learning [32]. Therefore, if researchers and teachers can provide the effective conditions and guidance to young children to promote their early metacognitive development, it will certainly lay a solid foundation for their good academic and developmental outcomes in the future.

### 1.3. Metacognition Intervention Approaches

In summary, reflective dialogue, visualized records, and play are three important approaches to develop the metacognitive skills of young children.

#### 1.3.1. Reflective Dialogues

Dialogic teaching or reflective dialogue between teachers and students as well as between students is considered to be a key approach to developing metacognitive skills [33]. Reflective dialogue between adults and children can encourage children to express knowledge of cognition [34]. The “Visual Learning” program requires teachers to guide students to make their thought processes clearer through the language of thought in their daily instruction to improve the students’ metacognitive skills [35]. Helping students clarify concepts, creating opportunities to speak, facilitating metacognitive talk among students, and creating conceptual conflict can improve the metacognitive skills of grade 1–6 students [36]. Creating situations, setting tasks, and asking questions also promoted the metacognition of young children’s [23].

#### 1.3.2. Observation Records

Constrained by the level of young children’s language and cognition development, there are specificities in teaching metacognition to young children. For example, the early development of metacognitive skills is underestimated due to the reliance on the children’s verbal self-reports [18]. Thus, in addition to abstract reflective dialogue, interventions for young children’s metacognition need to provide concrete materials to support them.

Recording the learning processes of young children and providing those records (e.g., photographs, videos) during reflective dialogue with young children could facilitate the children’s memory and metacognitive thinking. Reflective questions elicited extensive metacognitive thinking, encouraged children to reflect on their strategies for performing the task, and stimulated the self-interpretation of self-regulation and metacognition [37]. Pedagogical documentation supports the memory of children, offering them the opportunity to retrace their processes, find confirmation of negation, and to self-correct [38]. Children’s words, photographs, etc. enable them to clearly articulate their experiences and teachers and children can use observation recording tools and these visual records to improve the quality of discussions and reflections [39]. Anji Play also views the process of teachers observing and photographing, recording children’s play, and then organizing group or class sharing with children as a complex process that can facilitate reflection [3].

#### 1.3.3. Games

Play can promote young children’s related abilities of metacognition. An early education play curriculum named Tools of the Mind help children form play themes and plan their play in this curriculum. Teachers carefully monitor the play progress and guide children who may need help. Results suggest that preschoolers who received the program performed significantly better than the control children in inhibition, working memory, and cognitive flexibility [40]. Participation in adult-directed play helped preschoolers develop private language [41]. Rule-based play can significantly improve both the inhibitory control and working memory of 4-year-old children [42], which are related abilities of metacognition.

Based on the review above, intervention studies on the metacognition of children have mainly focused on students who finished their kindergarten education (i.e., 7-year old and above), while fewer studies have focused on children in the kindergarten stage and earlier (i.e., younger than 7-years old). It is necessary to incorporate these effective approaches into a metacognitive skills enhancement program and integrate them with daily teaching activities in Chinese kindergartens.

### 1.4. Anji Play Teaching Model

The Anji Play teaching model is well-suited for metacognitive enhancement activities. In the Anji Kindergarten curriculum system, its teaching and learning activities can be divided into three essential phases: play, reflection, and sharing, and follow-up activities.

First, children’s play is the starting point of teaching activities as play and teaching can transform each other, and teaching should come from play and promote play [43]. In the process of children’s play, teachers will observe and use photos or videos to record the problem-solving process or metacognitive performance of children. The ability to observe children is regarded as a core competency of teachers in Anji Play [44]. In addition to observation, teachers are also expected to record information about differences in the cognition ability development of children during play [45]. A report of the World Economic Forum in 2020 also highlighted the teacher’s supporting role in the observation of children’s interactions and problem-solving activities [46].

Second, after play and the teacher’s observations, Anji Play promotes reflection and sharing between teachers and students through the drawing of “play stories” by children, dialogue between teachers and children, etc. Through bold expressions and discussions, the critical thinking and logical expressions of children are greatly exercised in this process [47].

Third, children carry out follow-up activities on the basis of discussion such as starting a new play by drawing on the experience of their peers, or finding ways to verify guesses about the problem, or improving their own play on the basis of the original, or designing new play. In Anji Play, the children’s drawing works are viewed as “play stories”. First, children represent their direct experience in an abstract way through drawing. Then, children use language to abstract their experiences, and in this process, children can constantly review, reflect, and narrate their play experiences [3].

### 1.5. The Current Study

Based on prior studies, the current study designed the Circling Curriculum for Metacognition Training (CCMT), which integrates these three metacognition intervention approaches into ordinary kindergarten play activities. We also drew on the teaching model of Anji Play, highlighted the teacher’s observation and guidance role, create cognitive conflict situations and opportunities for individual inspiration for children, and allow children to develop metacognitive skills in an autonomous and voluntary play environment.

We implemented the CCMT to examine its effectiveness on promoting the development of metacognitions in 5–6 year old Chinese urban children. We intended to examine whether CCMT would significantly enhance the metacognition of the children who received the training. We expected that the CCMT would have a positive impact on the metacognitive skills of young children. Accordingly, we hypothesized that the experimental group would significantly outperform the control group in the post-test of metacognitive skills after early intervention.

## 2. Materials and Methods

### 2.1. Participants

A public kindergarten in Hangzhou of Zhejiang Province, China, agreed to participate in the current study. The researchers decided to use a medium effect size (effect size d = 0.5), two-sided testing, α = 0.05, β = 0.5, and an equal sample size in both groups. A power analysis with G*Power indicated that the sample size was 22, with power (1 − β) = 0.50. The experimental group (n = 25) and control group (n = 22) were therefore randomly selected from two kindergarten classes of 5- to 6-year-old children. The parents of every student in the two classes were invited and gave their approval to take part in this intervention study. Children in either group did not have any cognitive or learning issues that required clinician referral. Additionally, the independent *t*-test revealed no statistically significant difference in the demographic variables between groups (Table 1).

Furthermore, there were no significant between-group differences in the teachers’ qualifications and teaching experience. All four teachers held a Bachelor of Education degree, majoring in early childhood education. The class teachers in the experimental group and the control group had eight years and two years of teaching experience, respectively, and the assistant teachers in the experimental group and the control group had two years of teaching experience.

### 2.2. Design of the CCMT

According to Flavell et al.’s division of metacognitive dimensions, this intervention project divides metacognitive ability into the knowledge of cognition and the regulation of cognition and monitoring. Based on the Anji Play teaching model, each class includes three parts: before, during, and after the play, which involves the knowledge of cognition, on-line metacognitive, and off-line metacognitive thinking process.

**Figure 1:**
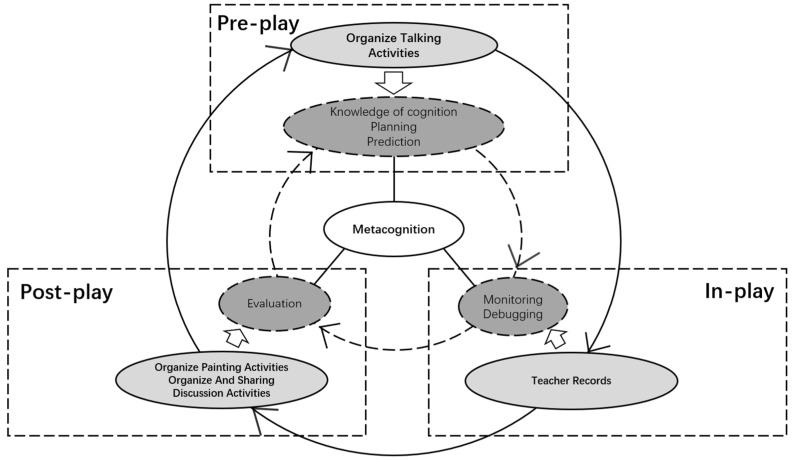
The framework of the Circling Curriculum for Metacognition Training.

The CCMT is a three-month intervention program. It includes three phases, with each phase lasting one month. For a detailed description of the aimed metacognitive content of each phase, the teaching objectives, and the contents in each stage, see Appendix A. The teaching framework of our CCMT is depicted in Figure 1.

There are two circles. Circle one shows the three dashed arrows form the Circling for children’s operation, and the dashed circle is the metacognitive skills used by the children in the corresponding session. Circle two: the solid arrows form the Circling for the teacher’s operation, and the solid circle is the teacher’s educational purpose in the corresponding session. The teaching strategies for each stage are depicted in Figure 1. For a detailed description of these strategies, see Appendix B. The overall framework of CCMT exhibited the following features.

#### 2.2.1. Playfulness

Children are born to enjoy playing. Most kindergartens in China have made independent play a regular daily activity, during which children play indoor and outdoor games.

Block play is a kind of construction play that combines small blocks into larger objects in a certain way to represent the physical world. It has long been considered as a major form of play in early childhood. Blocks are loose parts, which means that children can move, operate, control, and change during activities and can be used in a variety of ways and explore various possibilities. Block play can also stimulate multiple growth areas including language skills and social and cognitive development [48]. In addition, young children need autonomy to use different skills as they engage in block play. Block-building skills are considered as useful indicators of other abilities such as representational thinking [49]. Block play requires some knowledge and skills before playing, which can consolidate their understanding of the world around them. For example, children need to know the shapes of different buildings before they can build them while playing. The constructive nature of block play is conducive to the development of children’s logical thinking and reflection.

Therefore, we selected block play as the core activities in the CCMT. All interventions were related to the children’s block play process, and the children reflected and expressed themselves through the play process and various teacher-led activities.

#### 2.2.2. Systematic Applicability

This intervention program was conducted in a kindergarten class in Hangzhou, China, in the first semester of 2021–2022. The age range of the participating children was 5–6 years old. Chinese kindergartens usually conduct 1 to 2 h of play daily. The current study integrated the intervention program into the kindergarten’s daily play activities and provided a three-month systematic intervention for children. The intervention included two sessions a week, and each session lasted 1.5 h. The teaching session included three stages (i.e., pre-play, in-play, and post-play), and details are listed in Table 2.

#### 2.2.3. High Flexibility

The program is highly flexible, where each part is teacher-led but with children’s play as the main focus. It has fixed goals and activities, and the teachers’ guidance is adjusted according to children’s actual performance in the process of play. Children play independently according to their interests, and teachers provide timely feedback and support according to the children’s responses.

### 2.3. Instruments

#### 2.3.1. Knowledge of Cognition

We used the 12-item Metacognitive Knowledge Interview Questionnaire for young children [50] to measure the knowledge of cognition of 5–6 year old children in this study. Each dimension was measured by three items: Knowledge of persons (i.e., Item 1 to 3), knowledge of tasks (i.e., Item 4 to 6), and knowledge of strategies (i.e., Item 7 to 9). In addition, teachers rated the children’s answers on a four-point scale, ranging from zero to three (i.e., 0 = Not at all metacognitive, 1 = Partially metacognitive, 2 = Appropriately metacognitive, 3 = Abundantly metacognitive).

#### 2.3.2. Regulation of Cognition

We used the Metacognitive Monitoring Task [23] to measure the on-line metacognition, and the Metacognitive Skill Task [51] to measure the off-line metacognition of children. Those two measurements have been used in kindergarteners in China [23,24].

We provided children with two sets of puzzles and corresponding target pictures with two difficulty levels. The children were required to complete the puzzles according to the target picture. Before the task stars, the researcher asks the children Question 1 (i.e., How do you predict your performance?/Can you play it well?). After the task, the researcher asked the children Question 2 (i.e., How do you feel about your performance?/Did you play well?). The actual performance and answers to the performance-related questions of each child were recorded.

In the current study, planning refers to planning and thinking about how to play the game (e.g., what shapes of building blocks to use, the design of the building) to reach the goal of the game. Monitoring refers to the children’s monitoring of their play processes and manipulations including a comparison with the planning diagram and checking whether the blocks were built correctly. Debugging refers to the children’s behavior of regulating and correcting their own operations during play. The scoring criteria for on-line metacognition are as follows. Planning: The total time the researcher observed the participant stopped to plan and think before and during the operation. The duration was recorded in seconds. Each second was recorded as one point. Monitoring: The frequency that children observed the target picture during the operation. Each observation was scored as one point. Debugging: The frequency that the child removed a piece of a puzzle from work or returned a piece that was removed before. Each debugging action was scored as one point, and no points were awarded for only moving pieces of the puzzle.

In the current study, prediction refers to making predictions about whether one will be able to play the game and what level one will eventually achieve. Evaluation refers to the children’s evaluation of their level of play and the process of play after completing the game. The scoring criteria for off-line metacognition are as follows. The prediction score was rated on how the child’s actual performance was consistent with their prediction (i.e., answer of Question 1). The evaluation score was rated on how the child’s actual performance was consistent with their evaluation (i.e., answer of Question 2). These ratings used a three-point scale (0 = inconsistent, 1=basically consistent, 2 = completely consistent).

### 2.4. Procedure

The first author’s university reviewed and approved the study. We sent an invitation letter to the participating kindergarten, and the principal and the class teachers provided their consent to participate. We informed all of the parents about this study, and they consented to their children’s participation. All of the participating parents and kindergarten teachers signed written consent for this study. The participating kindergarteners verbally agreed to attend this study, and they knew that they could withdraw at any time.

The study included two parts. (1) Part one: Pre-experiment. We conducted the pre-experiment a week prior to the experiments. This aided the researchers in becoming familiar with the testing procedures, and guided the researcher to make necessary alterations to the training program. Ten 5–6 year old children (including five girls) participated in the pre-experiment. These ten kindergarteners were excluded in the formal experiment. We changed the puzzle’s difficulty level and the duration of the “in-play” stage based on the results of the pre-experiment. (2) Part two: Formal experiment. One week after the pre-test, the three-month Anji Play-based CCMT program was implemented. Metacognition learning activities was deliberately incorporated and elaborated in the experimental group, whereas the control group received routine courses with the same duration.

All of the children were administered the meta-cognition test before (i.e., pre-test) and one week after training (i.e., post-test). The same metacognition tasks were adopted in the pre-test and post-test phases to minimize the statistical deviation and the effects that such deviation might cause. In addition, we balanced the order of the metacognition tasks to avoid the possible effects of task order. We randomly used cross-coding to conduct the validity analysis.

### 2.5. Statistical Analyses

We computed the gain scores as the post-test score minus the pre-test score. We used SPSS version 22 (IBM Corp, Armonk, NY, USA) to conduct the statistical analyses. Using the Shapiro–Wilk tests, we checked the normality of the distributions of scores of the Metacognitive Knowledge Interview Questionnaire (score range: 0–3), the Metacognitive Monitoring Task (no upper limit to the score), and the Metacognitive Skill Task (score range: 0–2). Results indicated that the data were not normally distributed on these dimensions, *p*s < 0.05. Accordingly, we conducted a nonparametric Mann–Whitney U test to examine the differences between the experimental and control groups.

## 3. Results

The metacognition scores of the experimental and control groups before and after the intervention are presented in Table 3 and Table 4.

### 3.1. Testing Hypothesis

In order to examine whether the experimental and the control groups obviously differed in the pre-test, we conducted Mann–Whitney tests using the scores of all of the metacognition tasks. As given in Table 3, there were no significant differences among the experimental and control groups in all three dimensions of the knowledge of cognition, *p*s > 0.05. In addition, in all three dimensions of on-line metacognition and two dimensions of off-line metacognition, no significant differences were found between the experimental and control groups, *p*s > 0.05. In our summary, we found that all of the results on the pre-test set collectively showed no significant difference between the experimental and control groups.

The differences of all task scores between the experimental group and the control group in the post-test were calculated, respectively (see Table 4). Compared with the control group, the experimental group was higher in the total scores of the knowledge of cognition (*p* < 0.01), on-line metacognition (*p* < 0.05), and off-line metacognition (*p* < 0.05), with significant differences between the two groups.

The mean score of the knowledge of persons was 5.52 when the experimental group received CCMT and a mean score of 4.82 when the control group received routine teaching activities, constituting a significant difference (*p* < 0.05). The mean score of the knowledge of tasks was 5.60 when the experimental group received CCMT and the mean score of 4.77 when the control group received routine teaching activities, also constituting a significant difference (*p* < 0.05). In all three dimensions of on-line metacognition, children who received CCMT scored higher in planning (*p* < 0.05) and monitoring *(p* < 1.05) than the controls, constituting a significant difference. In two dimensions of off-line metacognition, the experimental group scored higher in evaluation (*p* < 0.05) than the controls.

Consequently, all of these results indicate significant differences in the post-test between the experimental and control groups, providing empirical evidence in support of our hypothesis.

### 3.2. Testing the Training Effects

In order to examine the training effects of the CCMT program, the gain scores of the experimental group and the control group were calculated, respectively (see Table 5). Higher gain scores indicate greater increases in metacognition abilities. We conducted Mann–Whitney tests on the gain scores between the experimental and control groups. In the knowledge of cognition (*p* < 0.01), on-line metacognition (*p* < 0.01), and off-line metacognition (*p* < 0.05), the total score of the experimental group was significantly higher than that of the control group.

For the specific sub-dimensions (see Table 5), compared with the control group, the experimental group showed higher gain scores of the knowledge of persons (*p* < 0.05) and knowledge of tasks (*p* < 0.05). In addition, in the planning and monitoring component of the on-line metacognition tasks, the gain scores of the experimental group and the control group were significantly different, and the experimental group was higher. Finally, for off-line metacognition in the dimension of evaluation, the gain score of the experimental group was also significantly higher than that of the control group.

In summary, all these results show that there was a significant difference in the gain scores between the experimental and control groups, demonstrating that the intervention program had a significant effect on improving the metacognitive abilities in the experimental group.

## 4. Discussion

As the first empirical study to explore the effects of a metacognitive enhancement program on the metacognitive abilities of young children in China, this study sought to evaluate the effectiveness a school-based metacognition enhancement program in 5–6 year old kindergarteners. The results support our hypothesis.

### 4.1. Effectiveness of the CCMT on Young Children’s Metacognition

Young children in the experimental and control groups did not exhibit significant difference in the pre-test. However, the experimental group children outperformed their counterparts in the control group on the metacognition scores in the post-test, and the gain scores of the total and most sub-dimensional scores. This result indicated the effectiveness of the CCMT on the metacognitive skills of young children.

The first feature of CCMT, likely related to its effectiveness, is the teacher-initiated interactions. During the implementation of the CCMT, teachers directly participate in and guide the cognitive process of the children, intervening in the pre-, in-, and post-play stages through questioning, dialogue, observation, and guidance. The results of previous studies on the role of adult involvement in the development of the metacognition of children are inconsistent. A study analyzed videos of teacher-initiated and child-initiated activities and transcripts of reflective conversations with children while watching the videos and found that the teacher could promote the development of the children’s metacognition, and teacher-initiated activity significantly impacted on how the children monitored their self-performance and learning [52]. However, another study suggested that young children tend to rely on adults when adults are present. In addition, when adults were absent, 4- to 5-year-old children exhibited better self-regulation and metacognition, particularly in the areas of goal setting, progress monitoring, and conflict resolution [53]. As the cognitive abilities of young children are still developing, they have difficulty actively shifting their attention from the play activity to metacognitive processes such as monitoring and regulation or allocating attention, much less than higher-order thinking activities such as reflection. Teacher guidance provides “scaffolding” to young children, especially in the areas of aim setting, progress monitoring, and strategy selection.

Another vital feature of the CCMT, the adaptation of Anji Play teaching model, may also contribute to its effectiveness. In the teaching process, teachers effectively listen, observe children, and record notes and photographs, reflecting that the learning experience is considered as an important way of supporting children’s self-regulation and metacognition [54,55]. Sharing activities with small groups allows children to revisit and reflect on their learning in an evidence-based way [31]. By using recording materials for reflective teaching, children can make reflections along with the adults and peers. Sharing activities allow children to observe the performance of others, listen to comments (and even critiques) of their peers and adults, and make comments on the scenarios presented by photo and video. Children’s drawing are inspired by what they feel, experience, know, understand, or imagine [56]. Through drawing, children can express something that they are unable to categorize or verbalize [57]. In the CCMT, the children’s drawing process is the process of externalizing their existing experiences. To map their problem-solving process, young children need to understand the mental concepts related to monitoring and regulation. The behaviors above-mentioned exemplify the various sub-dimensions of on-line and off-line metacognition [58]. Thus, this teaching process can strengthen the children’s related metacognition abilities.

Specifically speaking, children’s improvement in the knowledge of cognition may partially be due to the reflective dialogue. The current study found that the experimental group exhibited greater increases in the scores of the total knowledge of cognition and its two sub-dimensions (i.e., knowledge of persons and knowledge of tasks). The training activity begins with a reflective dialogue before play. Reflective dialogues promote children to use more knowledge of cognition. When critically examining self-regulation and metacognitive aspects of observing and recording, children have demonstrated significant knowledge of cognition indicators in reflective dialogue, specifically the knowledge of tasks and strategies [31]. Meanwhile, a study used video recordings of children’s activities and teacher–child reflective dialogues to analyze the children’s metacognitive behaviors and found that the knowledge of cognition was more frequently exhibited in teacher–child reflective dialogue sessions [51]. In the current study, teachers asked children questions (e.g., Do you like block play? How do you think you will do in the play, and why?) that were used to promote children to think about their cognitive abilities and previous experiences as well as the task at hand. These reflections are beneficial to the enrichment of the children’s knowledge of cognition. The reason for failing to find a significant group difference between the experimental and control groups in the knowledge of strategies may be due to the rapid development of the children’s strategic knowledge of cognition from 5 to 6 years old, as the age of 6 is a period of rapid development in the knowledge of strategies [24]. The strategic cognition of children in both groups may have been significantly improved. Thus, a group difference was not found.

These results provide empirical support for the effectiveness of CCMT in promoting the development of the regulation of cognition in young children. Children in the experimental group exhibited a higher level in the total on-line and off-line metacognition scores and in three out of five subdimensions (i.e., plaining, monitoring and evaluation). The possible reason for the children’s improvement in planning is that teachers provide children with planning opportunities in the pre-play stage, which encourages children to gradually develop from making loose and simple plans to making structured and complex plans. The plan formulated before play can also promote children to monitor their own play process, correct their play behavior, and make reasonable strategic choices. The evaluation may mainly improve through sharing and discussion activities. After play, the teacher would display photos and videos to help children review their actual play process and help children to identify the difference between their expected performance and actual performance. During the painting section, children review their play process and make reflections. Finally, in the discussion, the teacher provided the framework for evaluation by asking questions (e.g., Did you finished the task? Did the outcome different from your plan? Do you think you are doing well? Why?) to guide the children to express their evaluation process.

The reason for the lack of group difference in the debugging may be that after three months of intervention, the metacognitive abilities of children in the experimental class such as planning and monitoring had improved, and the analysis of problems had become more accurate, resulting in a reduction in the number of wrong behaviors in the Metacognitive Monitoring Task, and the number of times decreased. A previous study also indicated that when young children spend more time thinking, planning, and analyzing tasks and choosing effective strategies, the number of monitoring and debugging will be reduced [59]. In addition, the possible “ceiling effect” in prediction scores may, to some degree, account for not finding a group difference in this subdimension. As the M (SD) and max of prediction for the experimental group were 1.12 (0.73) and 2 in the pre-test, and their M (SD) and max reached 1.76 (0.66) and 3 in the post-test. This measurement defect should be corrected in future studies.

### 4.2. Educational Implications

The finding of this study clearly demonstrated that the 3-month CCMT can effectively enhance the metacognitive abilities of 5–6 year old children, which suggest that metacognitive abilities are teachable and can be enhanced in early childhood through well-designed interventions. It also sheds light on the early intervention in the metacognitive abilities of young children.

First, integrating the metacognitive intervention program into the kindergarten curriculum is a promising and worthwhile strategy. However, the intervention should be delivered appropriately to create a real space for metacognition-related education. Metacognition can be promoted by connecting other classroom activities in the curriculum and daily experience. It can help students to make sense of themselves and their world [60]. Kindergarten teachers can combine the teaching strategies in CCMT with daily activities (e.g., carrying out reflective dialogue in reading activities, encouraging children to draw the process after visiting the museum).

Second, metacognitive interventions need to be well-designed and flexible. Well-designed means that the interventions for the children’s metacognition should be closely integrated with the metacognitive process and inter-connected. For example, as metacognition knowledge enables children to plan, monitor, and evaluate their learning [61], the teaching of the knowledge of cognition should be arranged before play. Flexible means that teachers need to flexibly adjust strategies to each child and the specific play situation to maximize the effect of the intervention, for example, providing help to children with poor ability in the play and adjusting the order of questions in the reflection dialogue according to the children’s responses.

Finally, using a combination of photos and videos recorded by teachers and peer discussion can be effective in enhancing the metacognition of young children. The way teachers observe and record photos reflecting the learning experience and share will support the students’ self-esteem and learning awareness [55], which can enable children to demonstrate self-regulative and metacognitive abilities [62]. Photos and videos allow children to demonstrate their experiences to their peers. The narratives of the child help other children capture key information in pictures and videos more effectively. The display of the children’s experience with videos and photos as well as the summarized narratives of teachers are reviewed from other’s perspectives. Children review their play process from different perspectives, which are beneficial for the children’s reflection [3]. Our findings also suggest that in the discussion after presenting the photos and videos, young children show reflection on their own thinking (e.g., “I didn’t think so just now”, “I don’t know if I can use this tool”.). Allowing children to listen and to be heard through reciprocal interaction increased the children’s chances to practice evaluations [63]. This may shed light on future early intervention practices for metacognitive skills in authentic early childhood learning environments.

### 4.3. Limitations, Future Directions, and Conclusions

There are several limitations in this study that warrant further discussion. First, the CCMT was specifically designed for kindergarteners, but only 5–6 year old children in one urban public kindergarten participated in the current study. Future studies should utilize a sample with a broader age range (especially younger kindergarteners), larger sample size, and extended sociodemographic background to expand the sample’s breadth and increase the generalizability of the findings. Second, the current study did not collect follow-up test data of the children’s metacognition, which can examine the long-term effects of CCMT. In future studies, aside from the post-test, researchers should collect these data at intervals (e.g., two months) after CCMT implementation. Finally, the current study did not collect formal program fidelity data, which emphasize strict adherence to program design. The researcher implemented the intervention program, and to some degree, guaranteed its fidelity. However, future studies should collect these data, as these process evaluations must precede the outcome evaluations to provide the proper temporal sequence for evidence [64].

To conclude, despite these limitations, the present study provided important empirical insights into the metacognitive development of children aged 5–6 years. With ecological validity, the 3-month CCMT program demonstrated its effectiveness in enhancing the metacognition of young children in a real-world kindergarten context. The Circling Curriculum based on Anji Play, which includes reflective dialogue, the teachers’ effective observation records, and sharing and discussion activities, is an effective approach to promote this enhancement.

## Figures and Tables

**Table 1:** The demographic description for the experimental and control groups.

	Experimental Group (n = 25)	Control Group (n = 22)	*t*
Gender			
Boy	15	12	
Girl	10	10	
Age range (month)	60–73	60–75	
Age (month)	65.92 (3.58)	66.77 (3.87)	0.437

**Table 2:** Description of the three stages in the CCMT teaching framework.

Stage	Function	Duration
Pre-play	Conduct reflective dialogue between teachers and children, help children to enrich their knowledge of cognition, guide them to plan and predict, and provide a metacognitive foundation for the coming play activities	15 min
In-play	Observe and record the children’s regulation of cognition behavior during play	40 min
Post-play	Use various teaching strategies to help young children to recall and characterize cognitive processes to promote the improvement of evaluation and reflection skills	30 min

**Table 3:** The means, standard deviations, and maximum of the metacognition scores and Mann–Whitney test results in the pre-test.

	Experimental			Control			*u*	*p*
	M	SD	Max	M	SD	Max		
**Knowledge of cognition**	11.64	3.88	18	12.05	2.06	16	266.5	0.86
Knowledge of persons	4.48	1.66	7	4.36	1.29	6	253.5	0.63
Knowledge of tasks	4.16	1.57	6	4.36	1.46	6	254.5	0.65
Knowledge of strategies	3.00	1.44	6	3.32	1.49	6	240.5	0.45
**On-line metacognition**	33.32	10.60	53	33.00	11.30	52	274.5	0.99
Planning	9.36	6.40	26	8.68	7.67	29	234.5	0.39
Monitoring	18.12	6.59	36	17.59	8.80	35	272.0	0.95
Debugging	5.84	2.59	10	6.73	2.35	12	233.5	0.37
**Off-line metacognition**	2.12	1.42	5	2.00	0.87	4	269.5	0.90
Prediction	1.12	0.73	2	1.05	0.65	2	258.0	0.69
Evaluation	1.00	0.87	3	0.95	0.72	2	272.0	0.95

**Table 4:** The means, standard deviations, and maximum of the metacognition scores and Mann–Whitney test results in the post-test.

	Experimental			Control			*u*	*p*
	M	SD	Max	M	SD	Max		
Knowledge of cognition	15.56	2.26	20	13.73	1.83	16	121.0	0.00 **
Knowledge of persons	5.52	1.30	8	4.82	0.96	6	163.5	0.01 *
Knowledge of tasks	5.60	0.71	7	4.77	1.02	6	147.5	0.00 **
Knowledge of strategies	4.44	0.82	6	4.14	1.21	6	240.0	0.43
On-line metacognition	42.68	8.99	61	35.36	10.96	64	156.5	0.01 *
Planning	13.68	5.78	25	9.41	7.38	26	169.0	0.02 *
Monitoring	23.3	6..27	39	19.00	7.86	36	173.0	0.03 *
Debugging	5.68	1.49	9	6.96	2.79	12	206.0	0.14
Off-line metacognition	3.40	1.00	6	2.64	0.85	4	160.0	0.01 *
Prediction	1.76	0.66	3	1.46	0.60	2	215.5	0.16
Evaluation	1.64	0.70	3	1.18	0.73	2	189.0	0.046 *

Note. * *p* < 0.05, ** *p* < 0.01.

**Table 5:** The means (standard deviations) of the gain scores and Mann–Whitney test results.

	Experimental			Control			*u*	*p*
	M	SD	Max	M	SD	Max		
**Knowledge of cognition**	3.92	2.33	9	1.68	1.46	5	112.5	0.00 **
Knowledge of persons	1.04	0.98	3	0.45	0.91	3	174.5	0.02 *
Knowledge of tasks	1.44	1.39	4	0.41	1.14	3	156.5	0.01 *
Knowledge of strategies	1.44	1.29	5	0.82	1.10	3	198.5	0.09
**On-line metacognition**	9.36	4.79	21	2.36	5.43	12	92.0	0.00 **
Planning	4.32	3.54	11	0.73	3.03	8	120.0	0.00 **
Monitoring	5.20	3.73	12	1.41	3.76	10	123.5	0.00 *
Debugging	0.16	2.90	4	0.23	2.20	3	255.0	0.67
**Off-line metacognition**	1.28	1.10	3	0.64	0.66	2	179.5	0.03 *
Prediction	0.64	0.57	2	0.41	0.50	1	218.0	0.17
Evaluation	0.64	0.81	2	0.23	0.53	1	187.0	0.04 *

Note. * p < 0.05; ** p < 0.01.

## Data Availability

The data presented in this study are available on request from the corresponding author.

## Appendix A

**Table A1:** The overall structure of the Circling Curriculum for Metacognition Training.

Phase	Stage	Teaching Objectives	Teaching Content
**Phase One:** Enriching the knowledge of cognition	Pre-play	▪ Enriching knowledge of person and strategies	▪ Figure out one’s strengths and weaknesses in the play and possible reasons ▪ Extract and summarize the game strategies that have been used
	In-play	▪ Enriching knowledge of tasks	▪ Familiar with the rules and materials of block play ▪ Explore the relationship between the difficulty of the game and their own abilities
	Post-play	▪ Metacognitive experience preparation	▪ Recall the process of the play ▪ Describe the problems encountered in the play
**Phase Two:** Enhancing off-line metacognitive skills	Pre-play	▪ Developing metacognitive prediction skills	▪ Learning to use knowledge of cognition to make reasonable predictions
	In-play	▪ Transferring and applying knowledge of cognition	▪ Initially monitor the progress and completion of the play ▪ Choosing the right strategy based on monitoring results
	Post-play	▪ Developing evaluation abilities ▪ Foster the sense of evaluation	▪ Ability to represent the play process with drawings ▪ Learning the proper criteria for self-evaluation
**Phase Three:** Enhancing the regulation of cognition	Pre-play	▪ Reviewing knowledge of cognition ▪ Enhance Plan skills	▪ Learn to plan wisely ▪ Review previous play experience
	In-play	▪ Improving monitoring and regulation capacity	▪ Be able to adjust their behavior based on metacognitive monitoring results.
	Post-play	▪ Promoting metacognitive expression ▪ Improving evaluation skills	▪ Ability to clearly describe the problem-solving process ▪ Ability to conduct reasonable self-evaluation

## Appendix B

Description of teaching strategies.

### Appendix B.1. Talking Activities

Talking is a popular form of regular kindergarten activity, and guided conversations between teachers and children are considered as one of the most effective ways to improve the metacognitive skills of children. As 5–6-year-olds are still immature in their cognitive development, and their thinking is at the concrete image stage, they cannot make higher-level abstract generalizations about their thinking activities by themselves. Talking activities are equivalent to teachers providing “scaffolding support” and gradually guiding children to summarize and express their play processes and thinking processes by asking questions designed according to specific activities and the children’s age characteristics to externalize the implicit thinking processes and achieve the purpose of improving the metacognitive abilities of children.

### Appendix B.2. Teacher Records

During each independent play activity, the teacher observes the children’s play, capturing moments of metacognition in play. For example, during the construction process, the children always build against the plan; during play, the children say, “this round block will roll away” and “we are going to build a police station because the teacher introduced us to the police officer in the morning”.

### Appendix B.3. Painting Activities

According to Seitz, there are two ways of expressing the “primitive impulses” of human beings: words and drawing, thus visualizing inner experiences. Children naturally like to draw, and their drawings are the most natural expression of their thoughts and feelings. Therefore, in this study, after the independent play activities, the whole class was organized to draw their own “play stories” to record their play process, the difficulties they encountered in the game, and the strategies to solve them through drawing. Before the formal intervention, teachers guided the children to learn how to express their constructed play processes through drawing, providing representational symbols of characters, moods, and play processes.

### Appendix B.4. Sharing Discussion Activities

After the children have drawn their “play stories”, the teacher organizes a class discussion in which the children share their “play stories” voluntarily, introducing to the class the process of their play, the pictures they drew, and what they were thinking at the time. In this process, children recall their play and reflect on their cognitive activities. The teacher then shows the photos or videos they took based on their recordings of the day and askes the children in the photos or videos to share what they were doing at the time, using questions or dialogue as appropriate.
